# Topical Medications for Atopic Dermatitis and Effects on Increasing Lymphoma Risks

**DOI:** 10.7759/cureus.49135

**Published:** 2023-11-20

**Authors:** Brooke E Bocklud, Logan T Roberts, Dean T Roberts, Anne Schwartz, Harish Siddaiah, Shahab Ahmadzadeh, Giustino Varrassi, Sahar Shekoohi, Alan D Kaye

**Affiliations:** 1 School of Medicine, Louisiana State University Health Sciences Center (LSUHSC) Shreveport, Shreveport, USA; 2 School of Medicine, Louisiana State University Health Sciences Center (LSUHSC) New Orleans, New Orleans, USA; 3 College of Pharmacy, University of Louisiana Monroe, Monroe, USA; 4 Department of Anesthesiology, Louisiana State University Health Sciences Center (LSUHSC) Shreveport, Shreveport, USA; 5 Department of Pain Medicine, Paolo Procacci Foundation, Rome, ITA

**Keywords:** t-cell lymphoma, pimecrolimus, tacrolimus, calcineurin inhibitors, eczema, atopic dermatitis

## Abstract

Atopic dermatitis is an immune-mediated skin condition that causes relapsing, pruritic skin lesions. Flares of this disease are often treated with topical corticosteroids; however, the use of these drugs can cause unwanted side effects, such as cutaneous atrophy and impaired wound healing. To minimize these common side effects, severe forms of this disease have been treated with topical calcineurin inhibitors, which previously had no known long-term side effects. Recently, there has been debate on the immunosuppressive effects of these drugs and whether chronic use could result in non-melanoma skin cancer. Systemic absorption of topical calcineurin inhibitors is extremely limited compared to oral formulation, although it is directly proportional to the total body surface area applied with medication. Patients with atopic dermatitis can have an increased risk of lymphoma, so it is hard to distinguish the causative factor, e.g., severe atopic dermatitis or being treated with calcineurin inhibitors. While inconclusive, the Food and Drug Administration recently issued a black box warning, and currently, topical calcineurin inhibitors are considered a second-line treatment. The present investigation reviews the findings of multiple studies conducted to determine if there is a link between the usage of topical calcineurin inhibitors and lymphoma.

## Introduction and background

Atopic dermatitis (AD), commonly known as atopic eczema, is a chronic, relapsing dermatologic condition caused by an exaggerated immune response and dysfunctional skin barrier [1]. Traditionally, the mainstay of acute treatment has included routine use of topical moisturizers and corticosteroids (TCS) [2]. While effective for acute exacerbations, chronic use of TCS has many associated cutaneous side effects. Commonly encountered side effects include purpura, telangiectasia, striae, focal hypertrichosis, and acneiform or rosacea-like eruptions. Of greatest concern are cutaneous atrophy and impaired wound healing, which are problematic in a disease associated with an inadequate skin barrier. Related to these concerns, topical calcineurin inhibitors (TCI) were introduced in 2000 to be utilized chronically in patients two years and older with moderate-to-severe conditions [3]. The mechanism of these drugs is to inhibit an enzyme involved in cytokine production and T-cell activation. At the time of approval, no long-term safety studies had been conducted with tacrolimus, while pimecrolimus had been shown only to have an increased incidence of viral infections. Related to this lack of long-term safety data, the Food and Drug Administration (FDA) requested the manufacturers of both medications to conduct studies to prove their safety further. After completing a case-control epidemiological study, Novartis found no association between TCI exposure and non-melanoma skin cancer [4]. However, following this data, the FDA Pediatric Advisory Committee (PAC) met to review the findings. The meeting quickly shifted upon noticing an increasing off-label use of TCI in infants younger than two years of age and a few case reports of malignancies reported to the FDA's adverse event reporting system. In this regard, two registries were created to assess long-term safety further. The PAC assembled again in 2005, 15 months later, to re-assess their findings from the registries and a newly completed oral carcinogenicity study in monkeys. These findings indicated an association between malignancy and systemic exposure at a level high enough to illicit immunosuppression, which is not unexpected given the known association with systemic immunosuppression. There was no increased malignancy risk with lower systemic exposure levels, as seen with topical administration. Further complicated by increasing TCI prescription sales, persistent manufacturer marketing efforts, and elevated off-label use in infants, the committee's recommendation led to the FDA issuing a public health advisory and requiring a black box warning to address this theoretical lymphoma risk with topical administration [3]. Additionally, the indications were modified to emphasize that TCI are second-line therapy for acute and non-continuous chronic use [5]. There had previously never been a black box warning issued on a theoretical adverse event. The following hysteria that ensued saw prescription sales plummet, patient and prescriber concerns skyrocket, and the potential to properly conduct trials to prove the risks as participation in studies would be unlikely. Following surmounting criticism, numerous studies have been conducted over the years, hoping to dispel or provide discrete evidence to mitigate the inconclusiveness. Despite this, the FDA still has not altered its stance on the sliver of possibility for the potential association between TCI and lymphoma.

## Review

AD

AD is the most common chronic inflammatory skin disease [6]. Due to related immune dysfunction, it is commonly associated with other allergic conditions such as asthma and allergic rhinitis [1]. The onset of disease is typically within the first year and roughly 90% by the age of 5, though it may occur at any age. Clinical manifestations of AD include relapsing scaly lesions characterized by pruritis. The pathogenesis of AD is widely debated and multifactorial. Composed of multiple layers, the epidermis of the skin acts as a barrier to foreign substances to protect the host from exposure. The generally accepted etiology involves various mutations that result in a complex interaction between susceptibility genes and environmental triggers. For example, FLG gene mutations are considered a major risk factor in AD development because of the resulting skin barrier integrity demise following the loss of a key protein involved in epidermal differentiation. Additionally, other mutations have been identified that lead to dominance in pro-inflammatory cytokine-secreting Th2 cells and an unregulated expression of IgE molecules.

Combined, both components cause the patient to remain vulnerable to increased antigen exposures and dysfunctional response to antigens. Because AD is a relapsing condition with no cure, treatment is focused on alleviating symptoms. The first-line maintenance therapy includes the routine use of various emollients that aid in hydrating and fortifying the skin barrier against antigen exposure [7]. Emollients have been shown to improve skin appearance, dryness, and the need for pharmacological therapy. In acute flares, anti-inflammatory topical therapy can be used to induce remission. TCS have been the mainstay of therapy related to potent actions and accessibility. They act on a wide variety of inflammatory cells to quickly reduce inflammation. Dosing can be tailored since there is a range of available potencies, from lower-potency hydrocortisone formulations to high-potency clobetasol. Related to side effects and the need for alternatives in unresponsive patients, TCI initially became a popular second-line alternative to those ages two years and older with moderate-to-severe conditions that failed to respond to previous therapies. For comparison, tacrolimus 0.1% was found to be comparable in effectiveness to mid-potency TCS, while tacrolimus 0.03% was more effective than low-potency TCS formulations [7]. TCI poses the advantage of not causing cutaneous atrophy, which is particularly beneficial because of the already impaired epidermal layer of AD patients. Thus, clinicians recommend TCI over TCS when chronic intermittent treatment is indicated due to adverse effects associated with their long-term usage. Furthermore, TCS has been known to cause unpleasant cutaneous effects such as telangiectasia, so TCI are preferred in sensitive skin areas like the face, neck, or genitals.

Pharmacokinetics/pharmacodynamics of tacrolimus and pimecrolimus

Dysregulation of the function of T cells and increased IgE levels are characteristic of the pathogenesis of AD. Tacrolimus and pimecrolimus are among the calcineurin inhibitors that have been indicated as a treatment option for AD and solid organ transplantation prophylaxis [8]. Calcineurin inhibitors mechanistically act to inhibit the expression of interleukin-2 in T cells, which leads to the inhibition of inflammatory cytokine expression. Conventional tacrolimus oral formulation doses utilized in solid organ transplantation prophylaxis are dependent on the patient's body weight and adjusted based on systemic concentrations [9]. Tacrolimus exhibits poor oral bioavailability that is patient-specific and averages 25% bioavailability; however, the calcineurin inhibitor has a remarkable variation range of 5-90% bioavailability among all patients due to contributions from first-pass metabolism in the small intestine and liver. Extreme patient variability in bioavailability leverages increased difficulty to reach target blood concentrations of tacrolimus, which could ultimately lead to toxicity or rejection of the transplant. Nearly all (99%) of the systemically absorbed tacrolimus will bind to erythrocytes; however, only the dissociated portion of the drug is allowed to enter the lymphatic systemic to provide its immunosuppressive effects.

The primary driver in the metabolism of tacrolimus is the enzyme group CYP3A within the liver, which serves as a potential for numerous drug-drug interactions. P-glycoprotein (P-gp) further complicates the distribution and metabolism of tacrolimus through the inhibition of entry into organs or across the blood-brain barrier. Tacrolimus exhibits severely low clearance of 0.06 L/(h*kg)-1 with a variable half-life ranging from four to 41 hours and a mean half-life of approximately 12 hours. While tacrolimus is almost entirely (95%) excreted through biliary metabolism, urinary excretion accounts for 2%, and 0.5% of tacrolimus will be excreted unchanged through either feces or urine. As an immunosuppressive agent working to prevent graft rejections, tacrolimus exhibits high propensities for toxicity with a narrow therapeutic window, which suggests low exposure to tacrolimus may cause some patients to reject the graft and not others. Variability in the pharmacodynamics of tacrolimus can be attributed to several genetic polymorphisms, resulting in acute rejection, neurotoxicity, nephrotoxicity, delayed graft function (DGF), hypertension, and post-transplanted diabetes mellitus (PTDM).

To combat several IgE-mediated reactions associated with AD, calcineurin inhibitors are utilized as treatment options [10]. Calcineurin inhibitors, such as tacrolimus, are used topically as an alternative therapy to TCS to treat moderate-to-severe AD. Long-term use of TCS can be associated with adverse events such as dermal atrophy, allergic contact dermatitis, and rosacea. Though systemic tacrolimus exposure is severely limited compared to oral formulations, systemic blood concentrations proportionally increase as the total body surface area treated increases. Tacrolimus 0.1% and 0.03% are associated with blood concentrations ≤ 1 ng/mL and, often, unquantifiable systemic exposure measurements. Compared to patients being treated with systemic tacrolimus, patients receiving topical tacrolimus for AD exhibited negligible levels of systemic exposure [8]. The incidence of higher tacrolimus blood levels in patients using topical tacrolimus is considerably lower than in solid organ transplant recipients currently taking tacrolimus [11]. Pruritis and skin burning are the most frequently reported adverse events associated with the application of topical tacrolimus.

AD increasing the risk of lymphoma

Incidence rates of AD have increased over the past few decades, affecting approximately 20% of children in developed countries [12]. Patients diagnosed with AD often suffer from severe pruritis, which increases the risk for skin and soft tissue infections secondary to skin lesions. TCI are a cornerstone for the treatment of mild-to-severe AD. In pediatric patients ≤ 19 years of age, there is an incidence rate of lymphoma of one case per 100,000 persons in the United States; however, in patients ≥ 50 years of age, the incidence rate is 54 cases per 100,000 persons. Systemic immunosuppressive agents increase a patient's risk of developing lymphoma. Developing lymphoma is synonymous with intense immunosuppression while receiving solid organ transplantation and the immune system's inability to control Epstein-Barr viral infections. Those developing immunosuppression-related lymphoma most commonly develop B-cell non-Hodgkin's lymphoma, which may present as nodal or extra-nodal tumors and polymorphic. Both tacrolimus and pimecrolimus have been shown efficacious in the treatment of AD; however, neither immunomodulatory agents have been shown reflective of an increased risk of lymphoma [13].

The approval for TCI from the FDA was for the short-term or chronic intermittent treatment of AD, not the continuous, long-term therapy regimen, which garnered TCI a black box warning from the FDA regarding the potential associated risk of lymphoma. Long-term application of TCI tacrolimus or pimecrolimus in combination with TCS has observed a higher odds ratio (OR) in the development of lymphoma compared to monotherapy of either TCI or TCS [12]. The increased risk of developing lymphoma could be attributed to the continuous, long-term application of immunomodulating agents secondary to systemic immunosuppression.

AD severity has been closely entangled with an increased risk in the development of lymphoma, which is also attributable to other chronic inflammatory disease states such as rheumatoid arthritis and psoriasis. While it may be difficult to disseminate whether the disease state or the immunomodulatory agent is the cause for the development of lymphoma, increased severity associated with AD has a strong correlation with the association of heightened lymphoma risk compared to patients with a less severe disease state. There has been no substantial evidence that suggests that TCI such as tacrolimus and pimecrolimus significantly contribute to the development and/or overall risk of lymphoma.

Previous cohort studies and findings

There is a well-known debate over the use of TCI and the risk of lymphoma in patients with AD. On February 15, 2005, the FAD PAC put a black box warning on the use of pimecrolimus and tacrolimus in AD related to the potential risk of cancer. This black box warning was set due to post-marketing reports of malignancy in children and adults associated with the use of TCI [7]. Since then, numerous studies have been published with some supporting the causal relationship between TCI and lymphoma and others rejecting the association. In a retrospective cohort published in 2009, data was compiled from an integrated healthcare delivery system on 953,064 subjects diagnosed with AD or eczema between 2001 and 2004. In patients using tacrolimus compared to untreated patients, there was a significantly increased risk of cutaneous T-cell lymphoma in patients exposed to tacrolimus with a p-value of <0.001. The same was found in patients exposed to pimecrolimus and untreated patients, with a p-value of 0.010. No significant association was found for all other cancers. From these results, a conclusion was made that TCI increase the risk of cutaneous T-cell lymphoma only [14].

A systematic review and meta-analysis published in 2021 investigated the association between TCI and the risk of lymphoma in patients with cutaneous disease, using eligible studies from the date of inception to 2020. Pooled results showed that the use of topical tacrolimus and the risk of lymphoma had a relative risk of 1.68 with a 95% CI of 1.39-2.094. Results also showed that the use of topical pimecrolimus and the risk of lymphoma had a relative risk of 1.40 with a 95% CI of 1.13-1.74. TCI users had an incidence of lymphoma ranging from 0.02% to 0.09% compared to the control group with an incidence range of 0.02-0.06%. In a subgroup analysis, results showed that tacrolimus use had a relative risk of 1.89 with a 95% CI of 1.53-2.32 and pimecrolimus use had a relative risk of 1.38 with a 95% CI of 1.09-1.74 in developing non-Hodgkin's lymphoma. From this data, a conclusion was made that using topical tacrolimus or pimecrolimus significantly increased the risk of lymphoma, TCI users have a higher incidence rate of lymphoma, and TCI use is specifically associated with an increased risk of non-Hodgkin's lymphoma [15]. A European multicenter cohort study took place in 2018 comparing incidence rates of lymphoma and skin cancer in patients with AD using topical tacrolimus, topical pimecrolimus, and TCS and untreated patients. This study compiled 19,948 children and 66,127 adults treated with tacrolimus, 23,840 children and 37,417 adults treated with pimecrolimus, 584,121 patients treated with corticosteroids, and 257,074 untreated patients. The incidence rate ratio of lymphoma in patients treated with tacrolimus versus TCS was 3.74 with a 95% CI of 1.00-14.06 in children and 1.27 with a 95% CI of 0.94-1.71 in adults. When analyzing lymphoma type, the highest incidence rate ratio was 3.17 with a 95% CI of 0.58-17.23 for Hodgkin's lymphoma in children and 1.76 with a 95% CI of 0.81-3.79 for cutaneous T-cell lymphoma. A conclusion was made that there is a small increased risk of lymphoma in patients treated with TCI. However, this study acknowledged the possibility that AD itself is a risk factor for lymphoma and is therefore a confounding variable [16].

A cohort study published in 2009 used health insurance claims data adjudicated by medical records to compile patients who used topical pimecrolimus, topical tacrolimus, and TCS and patients with untreated dermatitis. Of 92,585 patients using topical pimecrolimus, 26 lymphomas were documented, with an incidence of 21/100,000 person-years. A similar incidence of lymphoma was found in patients using tacrolimus and corticosteroids. The relative risk of lymphoma in patients using topical tacrolimus was 1.16 with a 95% CI of 0.74-1.82. The relative risk of lymphoma in patients using TCS was 1.15 with a 95% CI of 0.49-2.72. This data indicates no significant increased risk of lymphoma in patients using topical pimecrolimus compared to topical tacrolimus and TCS. In addition, it was found that all three topical agents were associated with an increased risk of lymphoma compared to the general population, which can be attributed to increased detection of preexisting lymphomas [17]. A nested case-control study utilized the United Kingdom-based Health Improvement Network (THIN) database to access the risk of lymphoma in patients with AD using TCS or TCI. In this study, 2,738 cases of lymphoma were identified, and 10,949 matched controls. Results showed an OR of 1.83 with a 95% CI of 1.41-3.36 of lymphoma in patients with AD and an OR of 1.46 with a 95% CI of 1.33-1.61 of lymphoma in patients treated with TCS. Interestingly, no cases of lymphoma were found in patients treated with TCI. However, a conclusion was made that the sample size of patients treated with TCI was not sufficient to accurately study an association of increased risk of lymphoma [18].

A systemic literature search and meta-analysis published in 2015 compiled 24 references of case-control and cohort studies that discussed the risk of lymphoma and topical treatment in patients with AD. This systematic review contains the largest references and shows that the severity of AD itself is associated with an increased risk of lymphoma, while the association of TSC and TCI with lymphoma risk is not significant [13]. Overall, the use of TCI in AD has only been used in practice for a decade, limiting the ability to determine the long-term association between TCI and the risk of lymphoma. Existing data cannot exclude nor prove the association between TCI and increased risk of lymphoma [19] (Table 1).

**Table 1:** Articles investigating the association between TCI use in AD and increased risk of lymphoma TCI: topical calcineurin inhibitors; IR: incidence rates; TCS: topical corticosteroids; AD: atopic dermatitis

Author (year)	Groups studied and intervention	Results and findings	Conclusions
Study 1: Hui et al. (2009) [14]	A retrospective cohort study of the risk of lymphoma in 953,064 subjects treated with TCI for AD.	Increased risk of cutaneous T-cell lymphoma in patients using topical tacrolimus versus untreated patients (p-value <0.001) and in patients using topical pimecrolimus versus untreated patients (p-value 0.010).	TCI increase the risk of cutaneous T-cell lymphoma.
Study 2: Wu et al. (2021) [15]	Systematic review and meta-analysis of the association between TCI and risk of lymphoma in patients with AD.	The relative risk of lymphoma from topical tacrolimus use is 1.68 (95% CI of 1.39-2.094). The relative risk of lymphoma from topical pimecrolimus use is 1.40 (95% CI of 1.13-1.74). The incidence rate of lymphoma in TCI users is 0.02-0.09%. The incidence rate of lymphoma in the control group is 0.02-0.06%. The relative risk of non-Hodgkin's lymphoma in topical tacrolimus users is 1.89 (95% CI of 1.53-2.32) and 1.38 (95% CI of 1.09-1.74) in topical pimecrolimus users.	TCI significantly increase the risk of lymphoma, TCI users have a higher incidence rate of lymphoma, and TCI use is specifically associated with an increased risk of non-Hodgkin's lymphoma.
Study 3: Castellsague et al. (2018) [16]	A European multicenter cohort study comparing IR of lymphoma in patients with AD using topical tacrolimus (19,948 children; 66,127 adults), topical pimecrolimus (23,840 children; 37,417 adults), and TCS (584, 121 patients) and untreated patients (257,074 patients).	IR of lymphoma in patients treated with tacrolimus versus TCS is 3.74 (95% CI of 1.00-14.06) in children and 1.27 (95% CI of 0.94-1.71) in adults. When analyzing the type of lymphoma, the highest IR is 3.17 (95% CI of 0.58-17.23) for Hodgkin's lymphoma in children and 1.76 (95% CI of 0.81-3.79) for cutaneous T-cell lymphoma.	There is a small increased risk of lymphoma in patients treated with TCI. The diagnosis of AD itself is a risk factor for lymphoma and is a confounding variable.
Study 4: Schneeweiss et al. (2009) [17]	Cohort study using health insurance claims with medical records to find patients with AD and lymphoma and then determine if these patients used TCI or TCS or were untreated.	Of 92,585 patients using topical pimecrolimus, 26 lymphomas were documented, with an incidence of 21/100,000 person-years. The relative risk of lymphoma in patients using topical tacrolimus is 1.16 (95% CI of 0.74-1.82). The relative risk of lymphoma in patients using TCS is 1.15 (95% CI of 0.49-2.72).	TCI and TCS are both associated with an increased risk of lymphoma compared to the general population which is most likely due to the increased detection of preexisting lymphomas.
Study 5: Arellano et al. (2009) [18]	Nested case-control study using the United Kingdom-based Health Improvement Network (THIN) database to access the risk of lymphoma in patients with AD using TCS or TCI. 2,738 cases of lymphoma were identified and 10,949 matched controls.	There were an odds ratio of 1.83 (95% CI of 1.41-3.36) of lymphoma in patients with AD and an odds ratio of 1.46 (95% CI of 1.33-1.61) of lymphoma in patients treated with TCS. No cases of lymphoma were found in patients treated with TCI.	The sample size of patients treated with TCI was not sufficient to accurately study an association of increased risk of lymphoma.
Study 6: Legendre et al. (2015) [13]	A systemic literature search and meta-analysis of 24 references with case-control and cohort studies that discussed the risk of lymphoma and topical treatment in patients with AD.	The relative risk of lymphoma in cohort studies is 1.43 (95% CI of 1.12-1.81). The odds ratio of lymphoma in case-control studies is 1.18 (95% CI of 0.94-1.97). The severity of AD is a significant risk factor. Highly potent TCS are associated with an increased risk of lymphoma.	The severity of AD itself is associated with an increased risk of lymphoma, while the association of TCS and TCI with lymphoma risk is not significant.

## Conclusions

TCI are effective treatments for chronic intermittent AD. They were originally thought to have no long-term side effects making them an optimal treatment choice. However, a debate soon followed on whether these topical medications resulted in non-melanoma skin cancer. This inconclusively resulted in the FDA issuing a black box warning. There are several studies in this manuscript concluding that TCI increase the risk for lymphoma; however, the large-scale meta-analysis shows that the association of TCI with lymphoma risk is not significant.
